# Emotional responses to favorite and relaxing music predict music-induced hypoalgesia

**DOI:** 10.3389/fpain.2023.1210572

**Published:** 2023-10-25

**Authors:** Darius Valevicius, Anaïs Lépine Lopez, Ajar Diushekeeva, April Chaewon Lee, Mathieu Roy

**Affiliations:** ^1^ Roy Pain Imaging Lab, Department of Psychology, McGill University, Montréal, QC, Canada; ^2^ Alan Edwards Centre for Pain Research, McGill University, Montréal, QC, Canada

**Keywords:** music, pain, emotion, theme analysis, hypoalgesia

## Abstract

**Introduction:**

The hypoalgesic effect of music has long been established. However, the characteristics of music which are important for reducing pain have not been well-studied. Some research has compared subject-selected preferred music to unfamiliar music selected by researchers, and has typically found a superior effect from preferred music. In this study, we sought to discover what aspects of listeners' relationship with their preferred music was important in producing a hypoalgesic effect.

**Methods:**

We conducted a thermal pain and music listening experiment with 63 participants (14 male, 49 female, mean age = 21.3), in which music excerpts were paired with thermal stimulations. Pain ratings of intensity and unpleasantness, as well as emotional response variables, were rated on visual analog scales. We also conducted brief structured interviews about participants' favorite music, on which we conducted thematic content analysis. Themes and emotion variables were analyzed for their effects on pain ratings.

**Results:**

We first replicated the finding that favorite music outperforms experimenter-selected relaxing music in reducing pain unpleasantness (MD = −7.25, *p* < 0.001) and that the difference in hypoalgesia was partially mediated by an increase in musical chills (ab = −2.83, *p* < 0.01). We then conducted a theme analysis on the interview transcripts and produced four themes relating to emotional experience: *moving/bittersweet, calming/relaxing, happy/cheerful,* and *energizing/activating*. We found suggestive evidence that moving/bittersweet favorite music reduces pain unpleasantness through increased music pleasantness (ab = −5.48, *p* < 0.001) and more musical chills (ab = −0.57, *p* = 0.004).

**Discussion:**

We find that music pleasantness and musical chills are salient predictors of music-induced hypoalgesia, and that different categories of favorite music derived from qualitative analysis may engage these emotional pathways to different degrees.

## 1. Introduction

Music has been used to relieve pain for centuries, and in modern times, it has been found to reduce pain and anxiety in patients, as well as the need for medication (1–4). However, the mechanisms by which music reduces pain are not well understood (5). Some studies have indicated that subject-selected preferred music is more effective in reducing pain than experimenter-selected music (6, 7), but the structure of music preference and its contribution to pain relief have not been thoroughly examined.

Pain is a significant societal and individual burden, and there is a need for alternative ways to relieve it without over-reliance on pharmacological analgesics, which may produce side effects and dependencies (8–10). Music may be a viable non-pharmacological intervention for those undergoing surgery, surgical recovery, or with chronic pain conditions (11). To optimize music selection strategies for pain relief, research needs to identify the specific music attributes or emotional responses responsible for music-induced hypoalgesia.

One variety of music that is intuitively chosen in many experimental and clinical settings is relaxing music (6, 12, 13), but the effect of the level of relaxation in music on pain has not been systematically tested. Preliminary evidence suggests that relaxing music is better than stimulating music at relieving pain (7), but the low power in that study demands further investigation. One example of relaxing music that is already in use in clinical contexts is specially-composed relaxing music with a U- or L-shape of arousal, such as music produced by the MUSIC CARE app (12, 13). These instrumental tracks are composed in a variety of styles and genres, but possess a characteristic shape of arousal and tempo, where the tracks begin with a higher speed and energy before attempting to induce a state of deeper relaxation by transitioning to a slow, low-energy stage.

Some evidence suggests that subject-selected preferred music has a superior effect on pain relief regardless of the level of arousal in the music. Roy et al. (14) showed that for an equivalent level of arousal, pleasant consonant music reduced pain, while unpleasant dissonant music did not. In another experiment, Mitchell and McDonald (6) compared the effects of experimenter-selected relaxing music and subjects' preferred music on a cold pressor task. They found that only preferred music was able to reduce the intensity of pain, suggesting that relaxation in music might not be sufficient for hypoalgesia. Thus, in this study, we wanted to more deeply investigate the contribution of preference and emotion to music-induced hypoalgesia.

However, there are several ways of approaching music preference when selecting music for a pain relief study. One approach would be to present participants with several options of songs, of which they can choose the most pleasant (15). Another method is to allow participants to bring their all-time favorite music to the study, which incorporates additional aspects of preference such as familiarity, episodic memory associations, and individualized semantic meaning (16). More recent brain imaging studies (17) have opted to do this to ensure the most robust activation of brain structures related to processing music-related reward. However, the richness of different emotions, associations, and meanings that are involved in the experience of listening to one's favorite music has not been well-studied, particularly in the context of pain relief.

In this study, we sought to discover which aspects of the subjective experience of listening to favorite and relaxing music were particularly important for producing a hypoalgesic effect. We used a hybrid approach to this question, using a combination of qualitative and quantitative analyses. We invited 63 participants to come to the Roy pain laboratory on McGill campus to listen to relaxing and favorite music, as well as scrambled and silent controls, while receiving thermal stimulations. On the qualitative side, we conducted brief structured interviews with participants about their favorite songs and conducted a theme analysis (18) to categorize the content of these interviews. Four themes related to categories of emotional experience: *happy/cheerful, calming/relaxing, energizing/activating, and moving/bittersweet*. On the quantitative side, we examined the effects of several emotion variables on reducing pain, including music pleasantness, emotional arousal, and the incidence of “chills, thrills, or frissons”, and whether these could explain differences in pain ratings between favorite and relaxing music, and differences in hypoalgesia associated with emotional themes.

## 2. Materials and methods

### 2.1. Participants

63 healthy participants were recruited for this study (14 male, 49 female; mean age = 21.3, SD = 2.1) (see Table 1). Participants were recruited through advertisements posted on Facebook and through an extra credit system in the McGill department of Psychology. Criteria for exclusion from the studies included a history or current diagnosis of neurological or psychiatric disorder, diagnosis of chronic pain syndrome or neuropathy, history of alcohol or substance abuse, and regular (>2 weekly) use of analgesics, anticonvulsants, narcotics, antidepressants, and anxiolytics. Participants received either monetary compensation or course credits for their time. Informed consent was obtained from all participants and the study was approved by the McGill University Research Ethics Board.

**Table 1:** Age and gender properties of the study sample.

	*N*	Mean	Median	SD
Male	14	22.3	22	1.84
Female	49	21.0	21	2.04
Total	63	21.3	21	2.07

### 2.2. Stimuli

#### 2.2.1. Thermal stimuli

Painful thermal stimuli were induced by applying a 9 cm^2^ thermal contact probe (TSA-II Neurosensory Analyzer, Medoc LTD. Advanced Medical Systems, Israel) to the surface of the left inner forearm. This device has a 3 × 3 cm head which can output and maintain temperatures accurate to one decimal place. The sensation induced by the probe may be compared to a hot cup of coffee held against the skin. At the temperatures (<49.5°C) and time durations (10 s at plateau) at which this was done, there was no risk of physical harm to participants. The stimulations alternated between four different locations on the inner arm, where the ordering of locations was pseudo-random, with no stimulation of the same spot twice in a row.

#### 2.2.2. Music

Music tracks for the active conditions were obtained in the following ways: (1) The participant's favorite music was selected by the participants themselves, and could come from any source, with the only requirement being that they were at least 3 min and 20 s in length. Participants were asked to select two tracks that represented “their favorite music of all time”, and “the songs that they would bring with them to a desert island”; (2) The relaxing tracks were provided by the MUSIC CARE company (12) and cut to a length of 6 m 40 s, which contained a transition from a medium level of arousal to a low level of arousal (“L-shape” of arousal). Before the main procedure, participants could select between 7 tracks and could listen to 20-s samples to help them make their decision. The tracks included were “Cotton Blues”, “Jamaicare”, “Légende Celtique”, “Musique de Film”, “Nuit Cubaine”, “Reggae Calédonien”, and “Sega Mizik Kèr”.

#### 2.2.3. Controls

Scrambled controls for preferred music and relaxing tracks were produced by applying a scrambling algorithm to the tracks in the active conditions. The algorithm consisted of cutting the tracks into 500 ms fragments which were then randomly shuffled, with a 100 ms crossfade applied between them. This condition was intended to control for general acoustic properties of music (e.g., loudness, frequency spectrum) while lacking the musical structure of the original tracks. Silent trials were also used as a control condition for music. They were held for the same length of time as the music trials, and participants were asked to maintain their focus on the computer monitor during silent trials.

### 2.3. Procedure

The experiment consisted of pairing painful thermal simulations with music excerpts. Before beginning the main procedure, a sensory calibration procedure was conducted to estimate an appropriate stimulation temperature for each participant, corresponding to a rating of 50 on a 100-point scale (0 = “Not painful at all”, 100 = “Extremely painful”). The calibration consisted of seven temperatures between 40°C and 49°C applied to each of four locations along the left inner forearm. For each stimulation, heat was applied for 15 s, with a 2.5-s rise and fall from a 32°C baseline and a 10-s plateau. After each stimulation, participants rated whether the stimulus was felt as (1) painful, or (2) warm, but not painful. If the stimulation was reported as painful, participants rated the intensity and unpleasantness of the pain on a 100-point visual analog scale. A generalized linear regression model was fitted to the calibration data in order to estimate a temperature corresponding to a pain rating of 50 out of 100, which was used for every stimulation of the main procedure.

**Figure 1:**
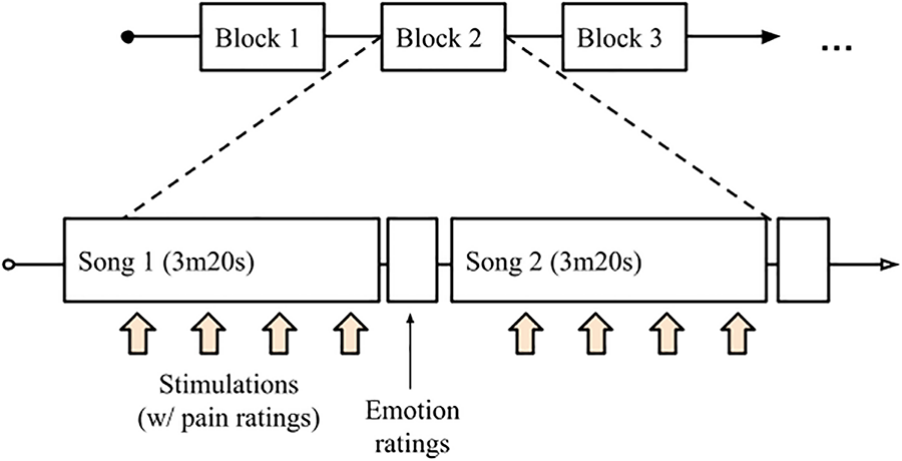
Structure of a block/condition. For favorite music and scrambled favorite music, two tracks of 3 m20 s were played; for MusicCare and scrambled MusicCare tracks, one longer track of 6 m40 s was played.

The main music listening task consisted of a series of approximately 7-min blocks (Figure 1). Each block represented a different condition. These were (1) Participant-selected favorite music, (2) Relaxing instrumental tracks, (3, 4) Scrambled versions of the favorite and relaxing music, and (5) silence^1^. The favorite music condition consisted of two songs played sequentially, each cut to a duration of 3 m20 s; the relaxing tracks, being longer, consisted of one track of 6 m 40 s. The order of conditions was randomized. For the task, participants wore a pair of high-quality, over-ear headphones (Audio Technica ATH-M50) and fixed their gaze on a point in the center of the monitor. Within each block, there were eight 50-s cycles of music and stimulation. The music, scrambled music, or silence was played alone for 35 s before the thermal stimulation was added for the final 15 s (2.5 s ramp-up and ramp-down with a 10-s plateau). After each stimulation, participants had 15 s to rate the intensity and unpleasantness of the pain they experienced, during which time the music continued uninterrupted. At the end of each track, participants rated the music's pleasantness, their emotional arousal, and the number of chills they experienced.

### 2.4. Measures

Quantitative variables were collected using visual analog scales (VAS) presented on a computer monitor, with anchors varying according to the variable measured. For pain, we collected two measures: (1) Pain intensity, representing the sensory dimension of pain, and (2) Pain unpleasantness, representing the affective dimension. A 0–100-point scale was used, with zero representing, e.g., “Not intense/unpleasant at all” and 100 representing “Extremely intense/unpleasant”.

Similarly, we collected measures of music pleasantness, emotional arousal, and the incidence of “chills, thrills, or frissons”. Music pleasantness was rated along a bipolar VAS, with −5 = “Extremely unpleasant”, zero = “Neither pleasant nor unpleasant”, and 5 = “Extremely pleasant”. Emotional arousal was rated along a unipolar scale with zero = “Not emotional arousing at all” and 10 = “Extremely emotionally arousing”. Finally, chills were measured using a four-point scale, with zero = “No chills at all”, 1 = “One or two chills”, 2 = “Three or four chills”, and 4 = “Five or more chills”.

### 2.5. Interviews

Participants were asked a series of open-ended questions about the favorite songs that they selected for the study. These consisted of three questions, asked separately for each of the two songs they selected. To assist participants in forming their answers, the songs were played in the background of the interview at a low volume. The interview took place at the end of the session. The questions are as follows:

(1) Why is this your favorite song, or why did you choose to bring this song? What do you like most about it?
(2) What thoughts, feelings, or images do you experience when you listen to this song?
(3) When do you listen to this song, or when do you find yourself wanting to listen to it?

The first question aimed to tap into what made the song salient to the participant. The second question focused on the content of the participant's experience when listening to the song. Finally, the third question was meant to tap into the function of the song, by asking participants what situations prompted them to listen to it.

### 2.6. Quantitative analysis

Linear regression analyses were performed using multilevel regression models using the R statistical programming language (19) and the lme4 package (20). Significance values were computed using the lmerTest package (21). Subject was used as a grouping factor for the intercept and all random effects. For specifying the random effects structures, we used a “keep it maximal” approach (22), including in the models any random effect term that did not interfere with model convergence. Figures were constructed using the R packages ggplot2 (23), sjPlot (24), and ggpubr (25).

For each analysis where pain was a dependent variable, several sources of nuisance variance were identified *a priori* and modeled using simple variables: (1) The trial number and (2) the log transform of the trial number were used to model sensitization and habituation, and (3) the location of the stimulation on the arm was included to model differences in mean pain between locations. Trial and log (trial) were *z*-scored and included as fixed and random effects as far as possible, and armspot was included as a grouping factor within subject. The noise models accounted for 8%–10% of the variance in both pain intensity and unpleasantness, in addition to the 46% accounted for by subject intercepts.

For testing the effects of categorical variables such as music conditions or favorite music themes, we used a dummy coding scheme (26), with the category of interest coded as one and the reference variable(s) coded as zero, and variables not of interest coded as NaN and thus excluded from the model. For a two-condition comparison (e.g., favorite music compared to scrambled favorite music), this resulted in a sample size of *n* = 980 and an effective sample size of approximately *n* = 128 after accounting for within-subject clustering of observations. This effective sample size was calculated using the intraclass correlation (ICC) observed for pain unpleasantness, which was 0.46.

Mediation analyses were conducted using the mediate package (27) with a simulation number of 500. Due to constraints on using the mediation package with multilevel regression models, the noise models (trial, log of trial, and armspot) were excluded from the mediation modeling.

Finally, in instances in this article where regression coefficients were converted into a standardized mean difference (SMD) or standardized effect size (d), we divided the coefficient by the average within-subject standard deviation (SD) for either pain unpleasantness or intensity (28). This value was 14.2 for both pain intensity and unpleasantness. Since all predictors were either dummy-coded or normalized, their standard deviations do not have to be accounted for.

### 2.7. Qualitative analysis

The qualitative interviews on subject-selected preferred music were analyzed using the theme analysis framework (18). Theme analysis requires researchers to consider their assumptions about the nature of the phenomenon they are categorizing. For this analysis, especially pertaining to themes describing emotional and psychological processes, we assumed a shared contribution of neuro-psychological realism and social construction, i.e., We assumed that emotional categories are based on evolved brain structures and functions and represent natural kinds to a certain degree (29, 30). However, the conceptual boundaries, nomenclature, and even the experience of these emotions are also dependent on cognitive, cultural, and linguistic factors (31). Therefore, we took a categorical approach to defining emotion themes, but allowed the interviewees' language to influence our categorization scheme rather than relying on a pre-defined theory or set of basic emotions.

Four researchers conducted the theme analysis. The process of determining the themes and sub-themes was conducted in an iterative and collaborative manner. Before constructing a list of codes, the researchers explored and familiarized themselves with the data and discussed the assumptions and goals of the analysis. Each researcher was assigned one half of the data to create a list of codes and themes, which were then integrated into a final list of codes through a series of discussions and revisions. The transcripts were then annotated using the final code list.

#### 2.7.1. Quantitative analysis of themes

Each theme was numerically coded into the data as either zero or one, representing its presence or absence in the interview response for that song. We examined whether the presence of absence of certain themes moderated the effect of preferred music on pain ratings. To do this, we added the dummy coded variables as covariates in a linear mixed model using only observations for the favorite music condition.

To attempt to give external validity to the emotion themes, we used a computational method for extracting musical features established by Fricke and colleagues (32, 33). This method uses the acoustic features of music and machine learning models to produce scores for different music dimensions, namely “arousal”, a dimension of intensity or excitement, “valence”, a dimension of happy to sad mood, and “depth”, a dimension of cognitive and emotional complexity in music (33). The dimensions were extracted for all favorite music tracks and correlated with the presence or absence of emotion themes using a simple linear regression model, to see if the themes extracted by reading structured interviews could be correlated with features derived from the audio waveform of the songs.

Finally, we correlated the incidence of emotion themes with personality variables collected per participant using simple Pearson correlation. We administered a short form of the Big Five Inventory (34), the Five Factor Mindfulness scales (35), the Musical Engagement Test (36), and the Pain Catastrophizing Scale (37). The results of this analysis are given in Supplementary Figure S1.

## 3. Results

### 3.1. Comparisons of active conditions with their respective controls

**Figure 2:**
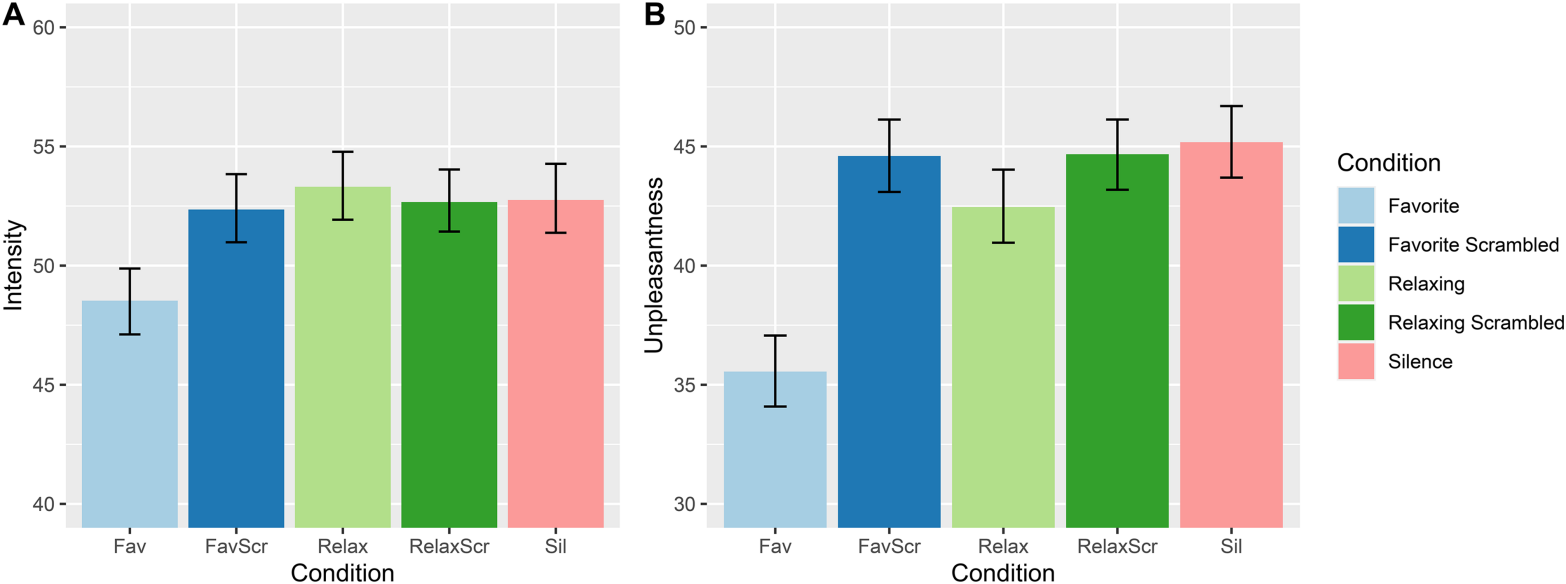
Means ratings of (**A**) pain intensity and (**B**) pain unpleasantness by condition, after controlling for between-subject mean pain ratings. Error bars = 95% CI.

We first examined whether the music condition could reduce pain intensity (INT) or pain unpleasantness (UNP) compared to their scrambled controls and silence (Figure 2). When compared to its scrambled control, favorite music reduced pain intensity (Mean Difference (MD) = −3.76, *t* (55.2) = −2.23, *p* = 0.030) and pain unpleasantness [MD = −9.05, *t* (57.9) = −4.62, *p* < 0.001], and also reduced pain when compared to silence (INT: MD = −5.14, *t* (155) = −3.71, *p* < 0.001; UNP: MD = −10.2, *t* (55.7) = −5.97, *p* < 0.001). Relaxing tracks did not significantly reduce pain intensity compared to their controls, however the effect on pain unpleasantness was trending towards significance when compared to scrambled music [MD = −2.51, *t* (55.4) = −1.34, *p* = 0.19] and silence [MD = −3.17, *t* (55.6) = −1.84, *p* = 0.071].

We also compared favorite and relaxing music to each other, and compared pain ratings between scrambled music and silence (Figure 2). We found that favorite music significantly reduced pain compared to relaxing tracks (INT: MD = −4.83, *t* (347) = −4.22, *p* < 0.001; UNP: MD = −7.25, *t* (53.8) = −4.81, *p* < 0.001). Meanwhile, none of the scrambled control conditions differed significantly from silence, with MDs under 0.7 and *p*-values above 0.6. The full results of the comparisons are given in Supplementary Table S1.

### 3.2. Effects of music pleasantness, emotional arousal, and chills

**Figure 3:**
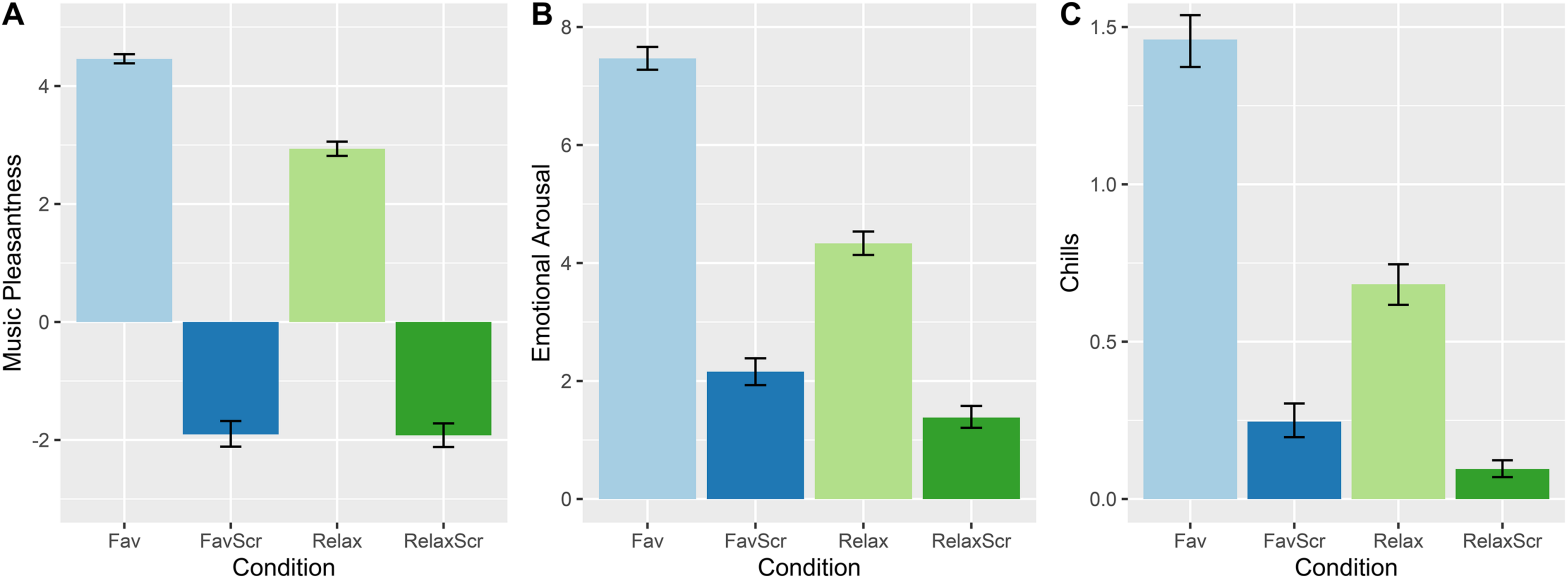
Mean ratings for (**A**) music pleasantness, (**B**) emotional arousal, and (**C**) chills across music and scrambled music conditions. See Methods (Section 2.4) for interpretation of y-axis values. Error bars = 95% CI.

Figure 3 compares the mean values for music pleasantness, emotional arousal, and chills across the different conditions. As expected, music was perceived as being more pleasant and produced more chills compared to scrambled controls. Additionally, subject-selected favorite music had higher average ratings on all three measures compared to relaxing tracks.

In assessing the effects of music-related emotion variables on pain ratings (Table 2), we found that the amount of chills reported influenced both pain intensity [*B* = −2.43, *t* (485) = −2.88, *p* < 0.01] and pain unpleasantness [*B* = −2.63, *t* (618) = −3.05, *p* < 0.01]. Meanwhile, music pleasantness (a proxy for emotional valence) did not significantly influence pain intensity [*B* = −2.40, *t* (45.5) = −0.80, *p* = 0.43], but had a large effect on pain unpleasantness [*B* = −8.74, *t* (57.7.) = −2.39, *p* = 0.02]. Ratings of emotional arousal did not influence either pain intensity nor unpleasantness (*B* < 0.4, *p* > 0.8).

**Table 2:** Effects of self-reported emotion variables on pain ratings.

Pain variable	Emotion variable	*B*	SE	df	*t*	*p*
Intensity	Music pleasantness	−2.40	3.00	45.5	−0.80	0.43
	Emotion arousal	−0.23	1.50	189.7	−0.16	0.88
	Chills	−2.43	0.84	485.5	−2.88	**0** **.** **0041**
Unpleasantness	Music pleasantness	−8.74	3.66	57.7	−2.39	**0**.**020**
	Emotion arousal	0.037	1.55	348.0	0.024	0.98
	Chills	−2.63	0.86	617.7	−3.051	**0**.**0024**

*B*, unstandardized beta, or points of pain on a 100-point scale per standard deviation change in the independent variable.

Bold indicates the significant *p*-value at *p* < 0.05.

**Figure 4:**
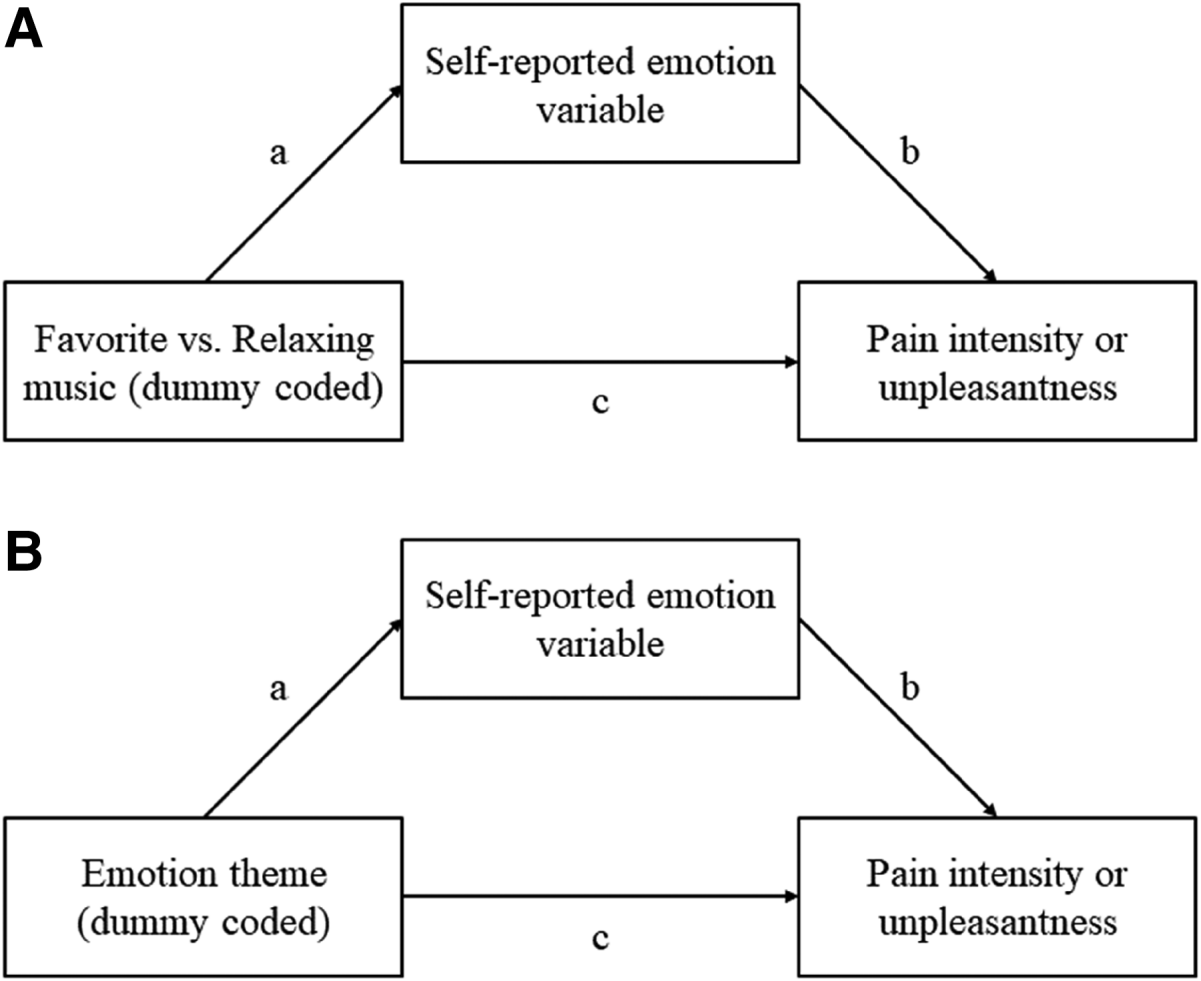
Mediation diagrams for analyzing (**A**) the difference between favorite and relaxing music, and (**B**) and the effects of emotion themes, using the three self-reported emotions variables as mediators: music pleasantness, emotional arousal, and chills.

We therefore conducted three mediation models (Figure 4A) to determine whether the difference between favorite music and relaxing music could be explained by emotion variables. Model 1 and Model 2 tested whether chills could explain the difference in pain intensity and pain unpleasantness respectively. Model 3 tested whether music pleasantness could explain the difference in pain unpleasantness. For Model 1, we found a significant indirect effect on pain intensity through chills [ab = −2.63, 95% CI = (−5.23, −0.39), *p* < 0.01] with a proportion mediated of 0.59. For Model 2, we also found a significant indirect effect on pain unpleasantness via chills [ab = −2.83, 95% CI = (−5.14, −1.01), *p* < 0.01] with a proportion mediated of 0.48. Finally, in Model 3, we failed to find a significant indirect effect on pain unpleasantness via music pleasantness [ab = −1.74, 95% CI = (−6.07, 2.88), *p* = 0.46, prop. mediated = 0.36]. One caveat in this analysis is that chills and music pleasantness are collinear, and thus the individual effects may be smaller than if one variable is included as a lone covariate. However, even after removing chills as a covariate from Model 3, we did not observe a significant mediation effect, though the effect was closer to the threshold of significance (*p* = 0.17).

### 3.3. Qualitative analysis of favorite music interviews

Participants were asked three open-ended questions for each of the two favorite songs they selected for the experiment (see Methods). A thematic content analysis was carried out which revealed 17 themes: two were centered on musical aspects, four on associations (to memories, persons, or imagery), four represented emotional categories, three were related to activities (e.g., commutes, tasks, or leisure), and four described listening times (e.g., morning, evening). The full list is summarized in Supplementary Table S2.

**Figure 5:**
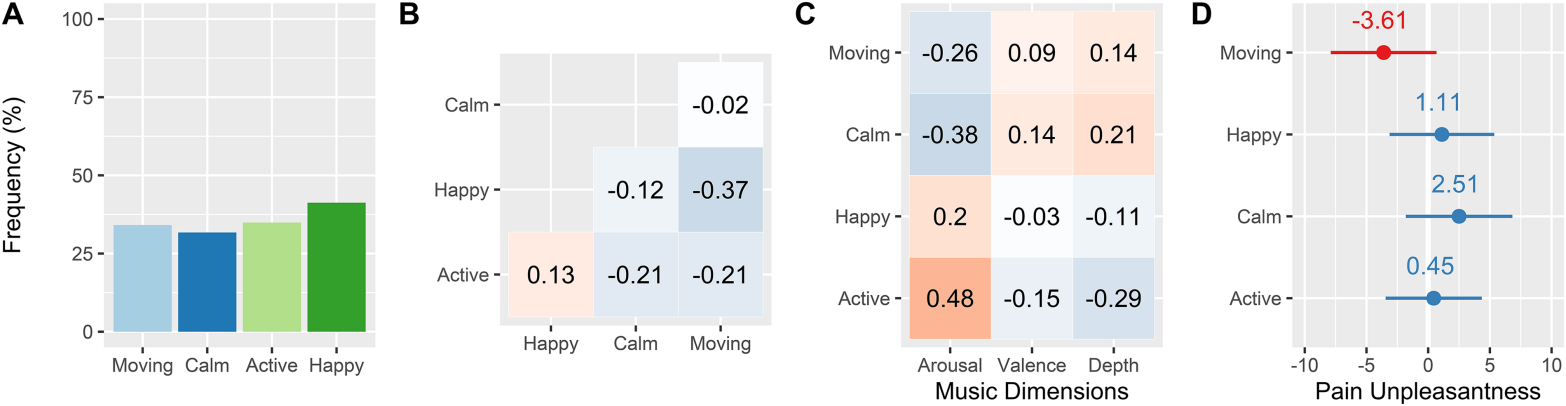
(**A**) Frequencies with which the emotion themes were reported in the study sample. (**B**) Intercorrelations between emotion themes. (**C**) Correlations between emotion themes and computer-extracted music dimensions. (**D**) Mean difference estimates of the pain-moderating effects of emotion themes. Error bars = 95% CI.

The four emotion themes—energizing/activating, happy/cheerful, calming/relaxing, and moving/bittersweet—are the focus of this analysis, as they formed a conceptually unified set of emotional categories and displayed greater correlations with external variables (e.g., computer-rated music dimensions) than the other themes (Quantitative results for the full set of themes are available in Supplementary Table S3). They appeared in roughly equal proportion in the interview data (32%–41% of responses for each theme; see Figure 4A) and show relatively low collinearity (Figure 5B), which facilitated their interpretation. The emotion themes are as follows:

#### 3.3.1. Energizing/activating

This theme encompasses descriptions of music such as “upbeat” (P02) and “pump-up” (P35). Participants mentioned that these songs gave them energy or raised their level of activation. For example, P05 mentioned wanting to listen to their song when they “want to be more ‘up, up, up’, when I need that energy…”. P10 said they would listen to their song “every time I need energy. […] Some people take coffee […] and I will listen to that song every time I need to get in the mood to do something that I don't really want to do”. “Power” was another common term. P57 described a song as, “Powerful. The beats are relatively quick. It's more of an aggressive song. […] It helps just empower you, I guess”.

#### 3.3.2. Happy/cheerful

The most frequent emotion theme was happiness. Very frequently, participants reported that their song made them happy (e.g., P24, “It really makes me happy”; P26, “I like this song because it makes me happy”). P27 reported that when they listened to their song, they experienced “just, like, happiness. I just want to bounce up and down. It just makes me smile”. Other common terms were “uplifting” (P27), “joy” (P29), and “fun” (P30). Some participants mentioned using their song to get them out of a negative mood, as P32 describes: “When I’m feeling distracted, and when I’m feeling sad about life, I just pull this out with the video, […] I have it saved permanently on the tab, and I feel better after.” Many participants simply said that “it makes me feel good” (P40) or that “it puts me in a really good mood” (P41).

#### 3.3.3. Calming/relaxing

This theme referred to songs that were described as calm or peaceful, or that participants used to calm themselves down or relax. For example, P05 said about their song, “This is the best song for me to be relieved […] I thought maybe this song would really help me to calm down.” P07 said of their song, “I like it because it's very mellow and chill”. P21 described experiencing “a feeling of ‘steady’, like everything is smooth.” Occasionally, participants paired the emotion with relaxing imagery, such as P34: “I just think about calmness and being on the beach”; or P05: “it's me walking along the riverside”. Other times, participants reported listening to their song when stressed or anxious, using it to lower their level of arousal, such as P51, “I [usually listen to this song] when I’m really stressed,” or P62, who listened to their song “mostly before competitions […] it relaxes my nerves. But I also listen to it before exams. I would say before going into something somewhat stressful.”

#### 3.3.4. Moving/bittersweet

This theme encompassed several sub-themes that were often difficult to categorize or describe. These songs did not seem to serve a concrete purpose, such as elevating mood, enhancing energy, or decreasing anxiety. Instead, the experience of varied and deep emotions seemed to be the end-in-itself. Many subjects described their songs as moving or emotional, and frequently referred to sad, bittersweet, or ambivalent feelings, such as P17: “This song in particular is super moving […] I just like it because it has so much emotion, but it's kind of negative emotion. Like passion, I guess.” P18 said of their song, “it's kind of sad, kind of emotional but it's also a really great song.” P19 said, “When I first listened to it, I got really emotional for some reason […] I started crying, and I think it has a really big emotional impact on that moment.” A common sub-theme was romance or love. P08 described that “the melody is something that makes me feel beautiful and loving.” P10 explained that “it's a love song, and it brings some feelings in me that I can't really describe.” Another sub-theme was one of resonance with a deep sense of self or significant personal meaning. P12 said, “I find a lot of connection with the meaning of the song. Just in my life in relation to other people, so it just feels like a very constant idea in my life.” P13: “I resonated with the message of it, so it's been important to me since it came out”. This theme seems to refer to music listening experiences that feature mixed or negative emotions, a strong sense of meaning, and sub-themes of love or romance.

### 3.4. Quantitative analysis of emotion themes

Computer-extracted arousal, valence, depth values were computed for all the favorite songs (Fricke et al. 2018), and Pearson correlations were computed between these dimensions and the presence of emotion themes (Figure 5C). Energizing/Activating correlated highly with high arousal (*r* = 0.47, *p* < 0.001) and low depth (*r* = −0.29, *p* < 0.001); calming/relaxing correlated with low arousal (*r* = −0.38, *p* < 0.001) and high depth (*r* = 0.21, *p* = 0.018); and moving/bittersweet correlated with low arousal (*r* = −0.26, *p* = 0.0039). Happy/cheerful displayed no significant correlations.

We next examined whether the emotion themes could modulate the hypoalgesic effect of favorite music (Figure 5D). Because of the relatively small number of observations per subject in this analysis (eight), and the low variation in emotion themes within subjects, the dummy variables representing emotion themes were not modeled as random effects. Of the four emotion themes, we found a near-significant effect of moving/bittersweet on pain unpleasantness ratings [MD = −3.61, *t* (482) = −1.65, *p* = 0.099]. The effect of calming/relaxing was not significant [MD = 2.51, *t* (482) = 1.14, *p* = 0.25], but possibly indicated a small increase in pain unpleasantness ratings. Happy/cheerful and energizing/activating showed no apparent effect (MD < 1.2, *p* > 0.6).

To see if the suggestive effects of emotion themes could be explained by subjective emotion variables (music pleasantness, emotional arousal, and chills), we conducted a second mediation analysis (Figure 4B). In examining whether emotion themes predicted the emotion variables, we found that moving/bittersweet significantly predicted music pleasantness [*B* = 0.44, *t* (483) = 5.69, *p* < 0.001], emotional arousal [*B* = 0.65, *t* (457) = 5.44, *p* < 0.001], and musical chills [*B* = 0.22, *t* (466) = 3.72, *p* < 0.001]. Meanwhile, a negative association with chills was observed for happy/cheerful [*B* = 0.22, *t* (469) = −3.76, *p* < 0.001], calming/relaxing [*B* = −0.28, *t* (467) = −4.87, *p* < 0.001], and energizing/activating music [*B* = −0.16, *t* (461) = −2.94, *p* = 0.0035]. No association with music pleasantness or emotional arousal was found for the latter three themes.

Importantly, our study design was not sufficiently powered to disentangle the question of whether the differences in emotion variables were specifically due to the category of favorite music, or due to individual differences in proneness to chills or emotional engagement in music that is associated with the choice of favorite music. It is likely that the effects are a combination of these two factors. To test this hypothesis, we performed a cursory analysis using the Musical Engagement Test (MET). We found that, though total MET scores significantly predicted chills [*B* = 0.30, *t* (60.4) = 3.07, *p* = 0.0033], including them as covariates in a multivariate model did not change the relationship between moving/bittersweet music and chills or music pleasantness.

We computed mediation models on the emotion themes using chills and music pleasantness as mediators, in order to see if indirect effects existed which could explain the observable differences in pain ratings between emotion themes. We found that the suggestive effect of moving/bittersweet on pain unpleasantness could be significantly explained by ratings of music pleasantness [ab = −5.48, 95% CI = (−8.96, −2.56), *p* < 0.001, Prop. mediated = 0.84] and by chills [ab = −0.57, 95% CI = (−1.14, −0.14), *p* = 0.004, Prop. mediated = 0.16]. For calming/relaxing music, we found a significant indirect effect through chills [ab = 0.87, 95% CI = (0.25, 1.80), *p* < 0.001, Prop. mediated = 0.21], in which the lower number of reported chills resulted in increased pain ratings. The same indirect effect through chills was observed for happy/cheerful [*B* = 0.61, 95% CI = (0.12, 1.19), *p* = 0.008, Prop. mediated = 0.06] and energizing/activating music [*B* = 0.43, 95% CI = (0.081, 0.91), *p* = 0.04], though these did not translate into any apparent total effect.

Finally, we correlated the reporting of emotion themes with personality variables: The Big Five Inventory, Five Factor Mindfulness Scales, Musical Engagement Test, and the Pain Catastrophizing scale. After applying a conservative alpha value (*ɑ* = 0.02, or a *p*-value threshold of 0.01) to adjust for multiple comparisons, an association between moving/bittersweet and affective, narrative, and social musical engagement existed (*p* < 0.01) and between energizing/activating and overall mindfulness (*p* < 0.01). At a more liberal alpha value of *ɑ* = 0.1, we found that moving/bittersweet was associated with greater BFI openness and lower FFMQ non-judging and non-reacting scores (*p* < 0.05), and energizing/activating was associated with lower pain catastrophizing and affective musical engagement (*p* < 0.05) The full results are given in Supplementary Figure S1.

## 4. Discussion

Few studies have compared the effects of different categories of music on pain, or gone into depth on the components of music that are effective in reducing pain. In this study, we compared participant-selected favorite music to experimenter-selected relaxing music created by the MUSIC CARE company (12, 13). We also conducted a hybrid analysis on the favorite music, using brief structured interviews and thematic analysis (18) to construct theme categories, and then investigated the relationships between emotion themes, self-reported emotional variables, and pain.

We first found that participant-selected favorite music strongly reduced pain intensity and unpleasantness compared to silent and scrambled controls, with an effect size of about 10 points on a 100-point scale for pain unpleasantness, or 0.7 standard deviations, and a smaller effect for intensity. Meanwhile, relaxing music was less effective in reducing pain, with an effect size of around 0.2 standard deviations on pain unpleasantness that did not reach statistical significance. One reason for this lack of a statistically significant effect of relaxing music may be a lack of power in our study design, which was estimated *post-hoc* at 0.63 for an effect of this size. However, our pattern of results does replicate previous research comparing preferred to relaxing music in an experimental pain paradigm (6), which also failed to show a significant effect of relaxing music on acute pain in a cold pressor task, which may indicate a general difficulty of achieving hypoalgesia from unfamiliar music in an experimental context.

In contrast, however, previous studies in clinical contexts have noted a significant effect of MUSIC CARE tracks on pain variables. A reason for this discrepancy may be that, due to experimental constraints, we were not able to present the tracks in the way they are intended to be used in a clinical setting (12). First, due to time constraints, we had to cut the 20-min tracks to about a 7-min window. In this window, we tried to include the transition from a higher-arousal starting tempo to the low-arousal middle part of the U-shaped arousal trajectory. In this way, we had some opportunity to induce the relaxed, low-arousal state that the tracks are intended to create. However, shortening both the induction part of the track and the low-arousal part may not have entrained participants to the same extent as the full track may have. A recent meta-analysis on music interventions in intensive care units has also shown that the length of the intervention is a critical component in pain relief, with interventions longer than 20 min showing a much larger effect than shorter interventions (38). In addition, our subjects were required to sit upright and attend to a computer monitor, which may not have allowed for the full induction of a relaxed state. Thus, the context and length of treatment may be an important component of leveraging relaxing music for pain relief.

After assessing the mean differences between relaxing music, favorite music, and controls, we examined the contribution of self-reported emotion variables. In conducting mediation analyses, we found that the incidence of musical chills significantly mediated the difference in pain ratings between relaxing and favorite music for both pain intensity and unpleasantness. Music pleasantness ratings, despite having a large direct effect on pain unpleasantness ratings, did not significantly explain the difference in pain scores between the two conditions. Self-reported ratings of emotional arousal did not affect either pain intensity nor unpleasantness. Thus, it seems that while both favorite and relaxing music judged as pleasant may be effective at reducing pain unpleasantness, there may be a neurophysiological process underlying chills which is preferentially recruited during favorite music that is more effective at reducing pain.

Neurological studies into music appreciation suggest that the mesolimbic dopamine pathway, including the nucleus accumbens (NAc), may be fundamental to both music enjoyment and music-induced chills (15, 17, 39, 40), and other studies have shown an association between NAc activation and pain perception (41, 42). One way in which activation of this pathway may alleviate pain is through the Motivation-Decision model (43, 44), where emotionally salient stimuli such as pain and music compete for conscious attention, possibly rooted in brain areas such as the insula and the anterior cingulate cortex (ACC), which are involved in interoceptive and emotional awareness. Another potential mechanism of music-induced hypoalgesia may be descending inhibitory pathways (41). The unique ability of chills to predict a reduction in pain intensity may point to this latter pathway being recruited during peak music listening experiences. Meanwhile, effects on pain unpleasantness may be primarily mediated by the former pathway, with the positive value of music competing with the negative value of pain when representing emotional states in conscious awareness. A recent fMRI study has provided suggestive evidence by finding a reduction in pain-related ACC activity during music listening (45). However, these hypothetical neurological mechanisms require substantial further study.

We next used a combination of qualitative and quantitative methods to analyze the effects of different aspects of listening to favorite music on pain perception. Using thematic content analysis, we extracted 17 themes from 126 brief structured interviews (two per participant). Four themes represented categories of emotional experience, which were the focus of our subsequent quantitative analyses. These were *happy/cheerful*, *energizing/activating*, *calming/relaxing*, and *moving/bittersweet*. Computer-extracted ratings of arousal, valence, and depth dimensions (32, 33, 46) provided some external validation of these categories, which had a variety of associations with arousal and depth (Figure 5C). The “moving/bittersweet” category is also comparable to the concept of “sweet sorrow” studied by contemporary researchers (47, 48), which involves the paradoxical appreciation many individuals have for sad music (49, 50).

We found suggestive evidence that the emotion themes differed in their ability to reduce pain, although with the relatively low power of this analysis, further research would be needed to confirm the existence of effects. We observed that moving/bittersweet was the strongest predictor of pain ratings, and showed indirect effects on pain unpleasantness via higher ratings of music pleasantness and musical chills. By comparison, calming/relaxing, happy/cheerful, and energizing/activating all showed lower levels of musical chills and significant indirect effects on pain unpleasantness, though significant total effects were not apparent.

Interestingly, calming/relaxing showed an opposite association with pain in comparison to moving/bittersweet, despite having a nearly identical computer-extracted feature profile, with lowered arousal and increased depth. Thus, it appears that a dimensional, music-centered approach such as the computer-extracted “Arousal, Valence, Depth” model (32, 33) may fail to account for certain experiences of music listening associated with mixed emotions or deeply meaningful experiences. This may highlight the need for more comprehensive subjective measures when studying music-induced hypoalgesia in future studies.

One issue with this analysis is that we were unable, due to limitations in our study design, to effectively disentangle the effects of emotion themes from individual differences in the average level of music appreciation and musical chills in participants who selected each type of favorite music. Thus, individuals who chose to bring moving/bittersweet songs may habitually experience more musical chills and musical enjoyment, resulting in lower pain ratings. However, a cursory analysis using scores from the Musical Engagement Test (36) failed to show evidence of this mediation dynamic. Future research could ask participants to bring songs from each category in order to tease apart these effects.

Finally, in assessing personality differences in participants who reported each theme, we found a significant association between moving/bittersweet and several aspects of musical engagement (affective, social, and narrative). We also found suggestive associations between moving/bittersweet and lower scores on the non-judging and non-reacting scales of the Five Factor Mindfulness scales (35), as well as higher scores on Big Five openness (34), indicating that participants who favor this theme may have a general tendency to engage more closely with their emotional experience, especially during music listening. Meanwhile, participants who reported the energizing/activating theme showed higher overall mindfulness scores, as well as suggestive associations with lower pain catastrophizing (37) and lower affective musical engagement. Further research could examine the links between personality and category of favorite music more closely.

Some limitations of our study include, as previously mentioned, that we did not use the relaxing tracks in their intended context. As such, the capacity of these tracks for reducing pain may be greater than what was suggested by our results, and may recruit additional mechanisms of hypoalgesia such as a hypnosis-like trance state (12). The results of our study are also limited to acute thermal pain in an experimental context, and further research is required to generalize it to clinical and chronic pain.

In terms of our qualitative analysis of themes in favorite music, our categorization scheme may be influenced by researcher biases and preconceptions. However, the varied associations with emotion variables, pain ratings, and computer-rated arousal, valence, and depth dimensions lend validity to these categories. The construct of moving/bittersweet also aligns with categorization schemes produced by other qualitative studies (47).

Finally, one potential limitation of the clinical use of favorite music for pain relief, particularly in an induced-pain or surgical context, may be that the negative aspects of the clinical experience may create aversive associations with the favorite music, reducing the pleasure individuals may take from it in the future. If this is the case, interventions would have to be selective or cautious about the use of favorite music for pain relief.

In conclusion, we find that favorite music is superior to experimenter-selected relaxing music in reducing acute thermal pain, and this difference is mediated by the strength of emotional responses to music, particularly the incidence of musical chills. In addition, the type of favorite music selected by the participant may modulate the effect on pain perception. Specifically, moving/bittersweet favorite music may be more effective in reducing pain due to increased music pleasantness ratings and musical chills. However, further research is needed to support these suggestive findings. Future research could also explore the neurobiological underpinnings of these effects, investigating in particular the roles of dopamine and the nucleus accumbens, as well as the insular and anterior cingulate cortex, in mediating the emotion-driven effects of music on pain.

## Data Availability

The datasets presented in this study can be found in online repositories. The names of the repository/repositories and accession number(s) can be found below: https://osf.io/m9xqd/.

## Footnotes

^1^Two additional conditions, consisting of unfamiliar popular music, were included to test a hypothesis relating to a separate study (Valevicius et al., in preparation), but are discarded from this analysis.

## Supplementary material

SUPPLEMENTARY FIGURE S1

Correlations between personality variables and emotion themes reported in participant interviews (*n* = 126). An X indicates a non-significant correlation at an alpha value of 0.10, or a *p*-value of 0.05. The correlations between Active and FFMQ Total, and between Moving and the MET scales, survived after applying a *p*-value cutoff of 0.01 to adjust for multiple comparisons. BFI, big five inventory; FFMQ, five factor mindfulness questionnaire; MET, music engagement test.

SUPPLEMENTARY TABLE S1

Mean comparisons of favorite music, relaxing music, silence, and scrambled conditions. MD, mean difference in pain points on a 100-point scale.

SUPPLEMENTARY TABLE S2

Full list of themes arrived at by thematic content analysis. Frequency refers to the proportion of the 126 interviews in the study that contained a reference to this theme.

SUPPLEMENTARY TABLE S3

Full regression results of emotion themes predicting pain unpleasantness ratings. One model was computed per theme category. MD, Mean difference in pain points on a 100-point scale when the theme is present vs. absent.
