# Supplementary figures and images for: Emotional responses to favorite and relaxing music predict music-induced hypoalgesia
Source: Front Pain Res (Lausanne). 2023 Oct 25;4:1210572. doi: 10.3389/fpain.2023.1210572 (PMC10630160; doi:10.3389/fpain.2023.1210572)

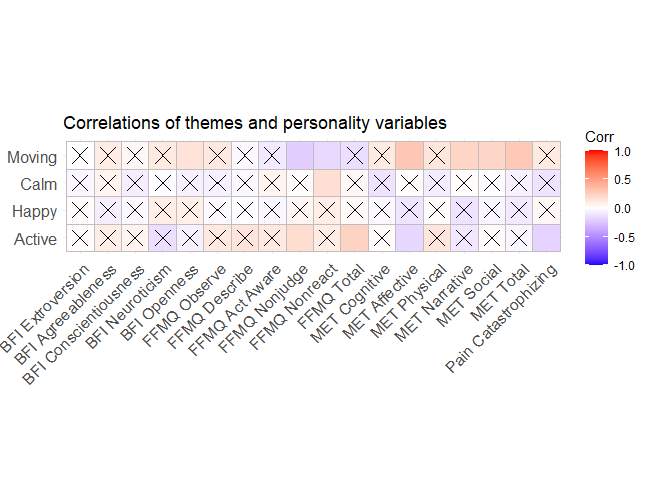

Supplement: Supplementary file 1 [file Image1.tiff]
