# Supplementary material for: Emotional responses to favorite and relaxing music predict music-induced hypoalgesia
Source: Front Pain Res (Lausanne). 2023 Oct 25;4:1210572. doi: 10.3389/fpain.2023.1210572 (PMC10630160; doi:10.3389/fpain.2023.1210572)
Supplement: Supplementary file 2 [file Datasheet1.docx]

**Supplementary Table 1.** Mean comparisons of favorite music, relaxing music, silence, and scrambled conditions. MD = Mean difference in pain points on a 100-point scale.

| Pain variable | Contrast | MD | SE | df | t | p |
| --- | --- | --- | --- | --- | --- | --- |
| Intensity | Favorite-Scrambled | -3.76 | 1.68 | 55.2 | -2.23 | **0.030** |
|  | Favorite-Silence | -5.14 | 1.39 | 154.8 | -3.71 | **0.00029** |
|  | Relaxing-Scrambled | -0.12 | 1.56 | 56.5 | -0.079 | 0.94 |
|  | Relaxing-Silence | 0.28 | 1.45 | 55.3 | 0.19 | 0.85 |
|  | Favorite-Relaxing | -4.83 | 1.14 | 347.1 | -4.22 | **< 0.001** |
|  | FaveScram-Silence | -0.60 | 1.23 | 139.9 | -0.49 | 0.63 |
|  | RelaxScram-Silence | -0.32 | 1.24 | 56.9 | -0.26 | 0.80 |
| Unpleasantness | Favorite-Scrambled | -9.05 | 1.96 | 57.9 | -4.62 | **< 0.001** |
|  | Favorite-Silence | -10.2 | 1.71 | 55.7 | -5.97 | **< 0.001** |
|  | Relaxing-Scrambled | -2.51 | 1.88 | 55.4 | -1.34 | 0.19 |
|  | Relaxing-Silence | -3.17 | 1.72 | 55.6 | -1.84 | *0.071* |
|  | Favorite-Relaxing | -7.25 | 1.51 | 53.8 | -4.81 | **< 0.001** |
|  | FaveScram-Silence | -0.42 | 1.40 | 55.3 | -0.30 | 0.77 |
|  | RelaxScram-Silence | -0.29 | 1.36 | 56.2 | -0.21 | 0.83 |

**Supplementary Table 2.** Full list of themes arrived at by thematic content analysis. Frequency refers to the proportion of the 126 interviews in the study that contained a reference to this theme.

| Category | Theme | Sub-themes (e.g.) | Freq. (%) |
| --- | --- | --- | --- |
| *Musical aspects* | Sonic/acoustic | Beat, melody, instrumentals | 59 |
|  | Narrative/lyrics | Story, imagined scenes | 41 |
| *Associations* | Attachments | Friends, family, partners | 30 |
|  | Memories | Events, places, nostalgia | 38 |
|  | Artist reference | Singer, album, cover art | 27 |
|  | Imagery | Colours, scenes, movie imagery | 21 |
| *Emotions* | Energizing/Activating | Upbeat, pump-up, power | 35 |
|  | Calming/Relaxing | For sleeping, when stressed | 32 |
|  | Happy/Cheerful | Fun, uplifting | 41 |
|  | Moving/Bittersweet | Emotional, romantic, meaningful | 34 |
| *Activities* | Performance/Participation | Singing along, dancing, playing on instrument | 17 |
|  | Ambient/passive | Commuting, hanging out, chores | 45 |
|  | Task focus | Studying, exercise, sports | 13 |
| *Listening times* | Morning | - | 11 |
|  | Evening/Night | - | 13 |
|  | Ubiquitous/All the time | - | 16 |
|  | Random/Sometimes | - | 8 |

**Supplementary Table 3.** Full regression results of emotion themes predicting pain unpleasantness ratings. One model was computed per theme category. MD = Mean difference in pain points on a 100-point scale when the theme is present vs. absent.

| Category | Theme | MD | SE | t | df | p |
| --- | --- | --- | --- | --- | --- | --- |
| *Emotion* | Moving | -3.61 | 2.18 | 482 | -1.65 | *0.099* |
|  | Happy | 1.11 | 2.16 | 481 | 0.52 | 0.61 |
|  | Calming | 2.51 | 2.2 | 482 | 1.14 | 0.25 |
|  | Active | 0.45 | 1.98 | 479 | 0.23 | 0.82 |
| *Musical aspects* | Sonic | -0.46 | 1.94 | 483 | -0.24 | 0.81 |
|  | Lyrics | 0.1 | 2.13 | 480 | 0.05 | 0.96 |
| *Associations* | Memory | -3.1 | 2.59 | 476 | -1.2 | 0.23 |
|  | Attachments | 3.86 | 2.58 | 481 | 1.49 | 0.14 |
|  | Artist | -1.32 | 2.18 | 481 | -0.6 | 0.55 |
|  | Imagery | 1.12 | 2.64 | 474 | 0.42 | 0.67 |
| *Activities* | Participation | -3.48 | 2.43 | 479 | -1.43 | 0.15 |
|  | Ambient | -4.24 | 2.11 | 478 | -2.01 | **0.045** |
|  | Task focus | 2.87 | 2.81 | 483 | 1.02 | 0.31 |
| *Time* | Morning | -3.94 | 3.34 | 465 | -1.18 | 0.24 |
|  | Random | 0.5 | 3.6 | 482 | 0.14 | 0.89 |
|  | Night | -0.71 | 2.89 | 479 | -0.25 | 0.81 |
|  | Ubiquitous | 0.31 | 2.87 | 475 | 0.11 | 0.91 |
